# Potential of natural flavonols and flavanones in the treatment of ulcerative colitis

**DOI:** 10.3389/fphar.2023.1120616

**Published:** 2023-03-03

**Authors:** Cailan Li, Ying Tang, Yonghao Ye, Manhua Zuo, Qiang Lu

**Affiliations:** ^1^ Department of Pharmacology, Zunyi Medical University, Zhuhai Campus, Zhuhai, China; ^2^ Key Laboratory of Basic Pharmacology of Ministry of Education and Joint International Research Laboratory of Ethnomedicine of Ministry of Education, Zunyi Medical University, Zunyi, China; ^3^ Key Laboratory of Basic Pharmacology of Guizhou Province and School of Pharmacy, Zunyi Medical University, Zunyi, China; ^4^ Zhuhai Resproly Pharmaceutical Technology Company Limited, Zhuhai, China; ^5^ Department of Nursing, Zunyi Medical University, Zhuhai Campus, Zhuhai, China; ^6^ Department of Pharmaceutical Sciences, Zunyi Medical University, Zhuhai Campus, Zhuhai, China

**Keywords:** ulcerative colitis, natural flavonols, natural flavanones, edible and medicinal plants, efficacy, mechanism

## Abstract

Ulcerative colitis (UC) is an inflammatory bowel disease generally characterized by chronic, persistent, recurrent, and non-specific ulcers of the intestine. Its main clinical manifestations include abdominal pain, diarrhea, and bloody stools. This disease is difficult to cure and even carries the risk of canceration. It has been listed as a modern refractory disease by the World Health Organization. Though a large amount of drugs are available for the inhibition of UC, the conventional treatment such as aminosalicylic acids, glucocorticoids, immunosuppressors, and biological agents possess certain limitations and serious side effects. Therefore, it is urgently needed for safe and effective drugs of UC, and natural-derived flavonols and flavanones showed tremendous potential. The present study concentrated on the progress of natural-derived flavonols and flavanones from edible and pharmaceutical plants for the remedy of UC over the last two decades. The potential pharmaceutical of natural-derived flavonols and flavanones against UC were closely connected with the modulation of gut microflora, gut barrier function, inflammatory reactions, oxidative stress, and apoptosis. The excellent efficacy and safety of natural flavonols and flavanones make them prospective drug candidates for UC suppression.

## Introduction

Ulcerative colitis (UC) is a non-specific chronic condition with pathological characteristics of inflammation and ulcer formation of the colorectal mucosa and submucosa (Lasa et al., 2022). Its clinical features are recurrent abdominal pain, diarrhoea, bloody stool, fatigue, and unexplained wasting, which are accompanied by different levels of complications in the skin, joints, mucosa, liver, lung, and other parts, bringing huge psychological distress and economic burden to patients (Li et al., 2021a; Kaluzna et al., 2022). With the continuous change of dietary pattern and structure, the incidence of UC in China is increasing year by year and presents an obvious trend toward younger age, and about 25% of UC patients are adolescents (Li et al., 2021b; Wang Y. F. et al., 2021). At present, UC has gradually become a public health challenge around the world.

The pathogenesis of UC has not been entirely defined. The current mechanism research mainly focuses on the complex role between genetic, immune, and environmental factors (Li et al., 2021c). This complex interaction ultimately triggered the activation of inflammation and the defect of epithelial barrier function, which are considered to be the central links in the pathogenesis of UC (Su et al., 2017). In the long-term chronic process of UC, the activation of mucosal inflammation and the impairment of epithelial barrier function jointly mediate a series of inflammatory cascades and the subsequent imbalance of intestinal microenvironment homeostasis and ultimately induce the generation of various disease states, including intestinal diseases and extra intestinal diseases (Li et al., 2020; Ferretti et al., 2022).

The establishment of an appropriate animal model is of great significance for exploring the developmental rules, pathogenesis, and preclinical drug screening of UC (Bilsborough et al., 2021). So far, there are six kinds of UC animal models: chemical stimulation, immune method, compound method, traditional Chinese medicine syndrome, gene modification, and spontaneous model, of which the chemical stimulation method is the most commonly used (do Nascimento et al., 2020; Eichele and Kharbanda, 2017). Chemical stimulation models include acetic acid, 2,4,6-trinitro-benzenesulfonic acid, oxazolone, 2,4-dinitrochlorobenzene-acetic acid, and dextran sodium sulfate models (Liu et al., 2020; Hu Y. et al., 2021). Among them, the histological and pathological changes of the UC model induced by dextran sodium sulfate are very similar to those of human UC, and it is currently recognized and widely used as an animal model (Hu et al., 2022).

**FIGURE 1:**
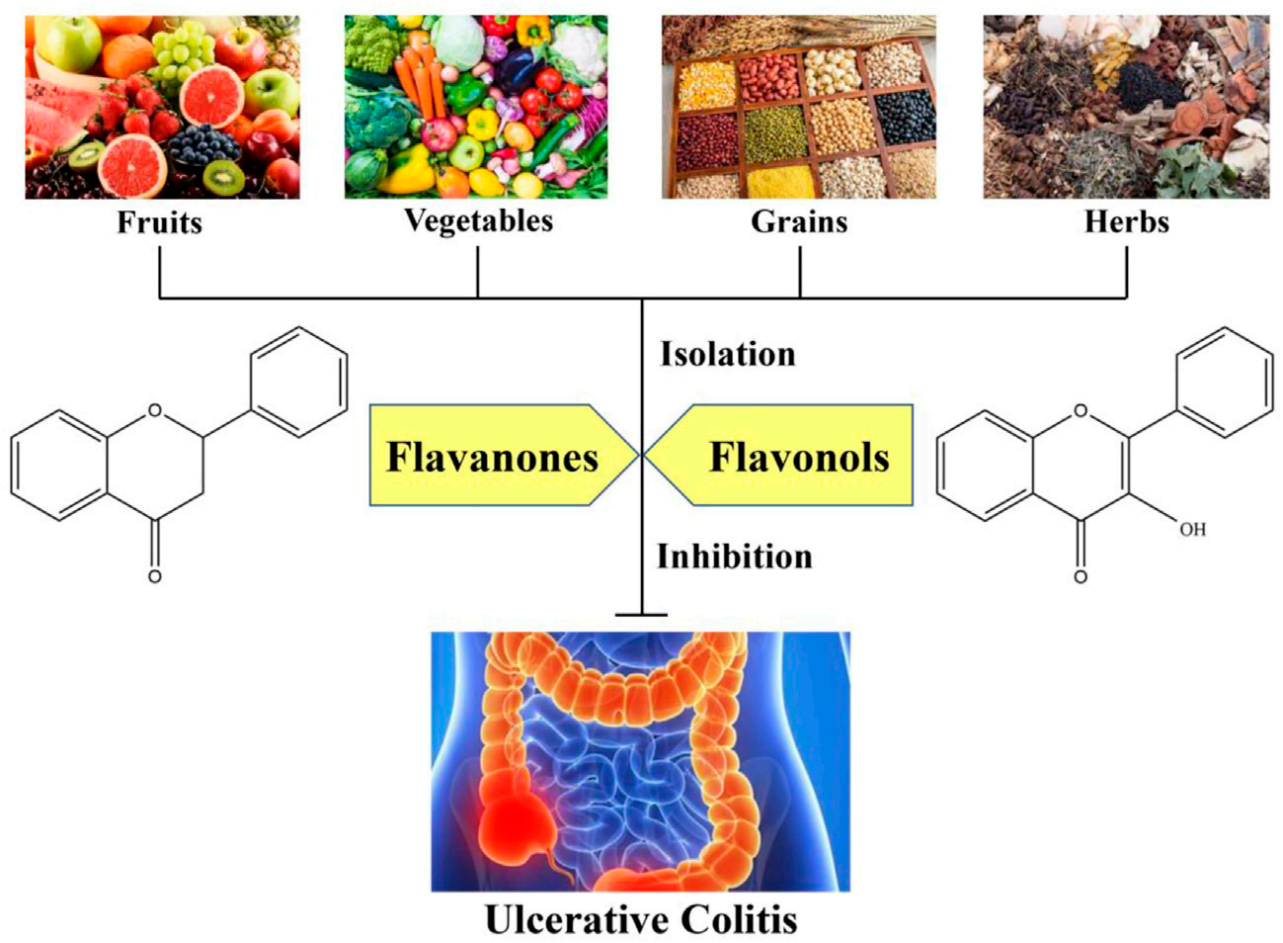
Natural sources of flavonols and flavanones against colitis in the past two decades.

Given that UC is a chronic condition with unknown etiology, its primary therapeutic goal is to treat and maintain clinical remission (Sinopoulou et al., 2021). In addition to diet control, the existing standard treatment mainly includes the use of aminosalicylic acids, glucocorticoids, immunosuppressors, and biologics (De Maio et al., 2022). However, the efficacy of these conventional treatment methods cannot meet the needs of patients. Meanwhile, the accompanying problems such as recurrence after drug withdrawal, hormone dependence and toxic and side effects also cause serious problems to clinicians and patients (Niu et al., 2021). It was reported that the cost-utility ratio of biologics in UC patients is increasing, and the annual cost of biologics can be as high as 450,000 dollars, which imposes a heavy economic burden on individuals, families, and society (Du and Ha, 2020). It is currently urgent to seek and explore more effective treatment strategies for UC. Plentiful studies have proved that many kinds of flavonols and flavanones (Figure 1) from edible and medicinal plants displayed prominent therapeutical potential on colitis. Therefore, this review summarized the progress of natural flavonols and flavanones in the treatment of colitis, which would offer novel perspectives for developing effective drugs for UC.

## Methods

To determine the studies connected with the effectiveness and mechanisms of natural flavonols and flavanones against UC, our team referred to the original articles in these databases from the inception date until September 2022: Web of Science, PubMed, Elsevier, Google Scholar, and CNKI. In the literature retrieval, the following key words were employed in combination: (“flavonol” OR “flavanone”) AND (“ulcerative colitis” OR “ulcer colitis” OR “UC”). All articles offering abstracts were taken into account.

After searching, the gained studies were severely filtered. First, the studies about the efficacy and mechanism of natural flavonols and flavanones against UC were selected based on title and abstract. Second, the entire text would be further checked for the studies that cannot be ascertained after primary filtering. Ultimately, all studies that conformed to the theme were collected and imported into EndNote as supportive resources for this review. The exclusion criteria were as below: crude extracts or oils, not natural-derived flavonols or flavanones, and combined treatments.

## Natural flavonols and flavanones against ulcerative colitis

**FIGURE 2:**
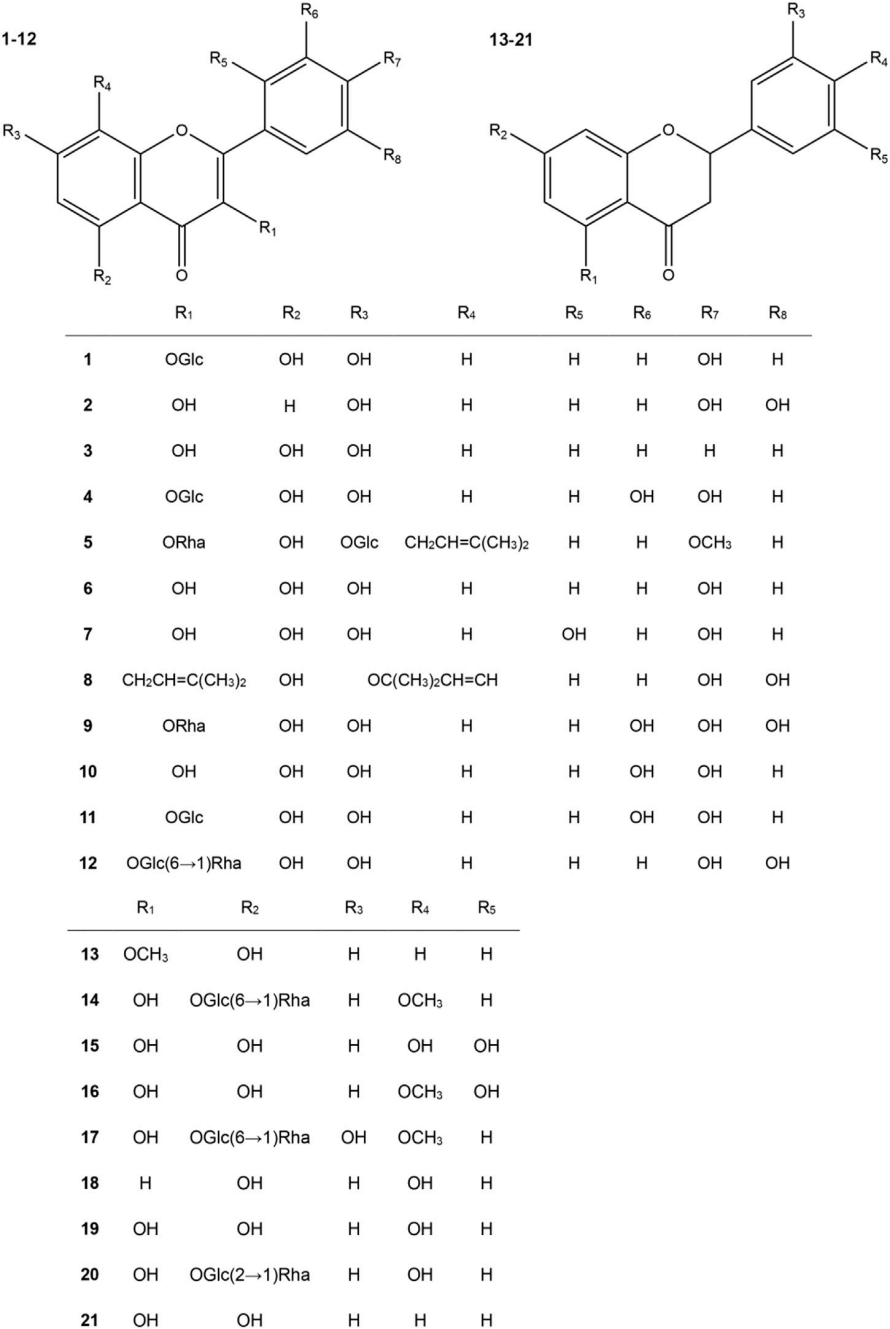
Structural formulas of natural flavonols and flavanones for the treatment of colitis.

Flavonoids are a large class of natural products used to treat colitis in plentiful studies. Among them, flavonols and flavanones have shown great potential and attracted the attention of many researchers. Therefore, this review systematacially exhibited the advances of natural flavonols and flavanones against UC. The general information on included flavonols and flavanones is displayed in Table 1, the chemical structure is exhibited in Figure 2, and the pharmacological information is showed in Table 2.

**TABLE 1:** Natural flavonols and flavanones extracted from edible and medicinal plants.

No.	Compounds	Molecular formula	Molecular weight (g/mol)	Main sources	References
Flavonols					
1	Astragalin	C_21_H_20_O_11_	448.38	*Astragalus membranaceus*; *Astragalus sinicus*; *Thesium chinense*; *Hippophae rhamnoides*	Han et al. (2021); Peng et al. (2020)
2	Fisetin	C_15_H_10_O_6_	286.24	*Fragaria ananassa*; *Toxicodendron vernicifluum*; *Rhus succedanea*	Sahu et al. (2016)
3	Galangin	C_15_H_10_O_5_	270.24	*Alpinia officinarum*	Gerges et al. (2020); Xuan et al. (2020)
4	Hyperoside	C_21_H_20_O_12_	464.38	*Hypericum perforatum*; *Crataegus pinnatifida*	Cheng et al. (2021)
5	Icariin	C_33_H_40_O_15_	676.66	*Epimedium brevicomu*; *Epimedium sagittatum*; *Epimedium pubescens*; *Epimedium koreanum*	Tao et al. (2013); Wang et al. (2016); Zhang et al. (2021)
6	Kaempferol	C_15_H_10_O_6_	286.24	*Kaempferia galanga*	Park et al. (2012); Qu et al. (2021)
7	Morin	C_15_H_10_O_7_	302.24	*Morus alba*; *Maclura cochinchinensis*	Gálvez et al. (2001); Ocete et al. (1998)
8	Morusin	C_25_H_24_O_6_	420.45	*Morus alba*; *Maclura cochinchinensis*	Vochyanova et al. (2017)
9	Myricitrin	C_21_H_20_O_12_	464.38	*Myrica rubra*; *Eugenia uniflora*; *Ampelopsis grossedentata*	Schwanke et al. (2013)
10	Quercetin	C_15_H_10_O_7_	302.24	*Fagopyrum esculentum*; *Allium cepa*; *Hippophae rhamnoides*; *Sophora japonica*	Dong et al. (2020a); Dong et al. (2020b); Ju et al. (2018); Lin et al. (2019)
11	Isoquercitrin	C_21_H_20_O_12_	464.38	*Fagopyrum esculentum*; *Allium cepa*; *Hippophae rhamnoides*; *Sophora japonica*	Cibicek et al. (2016)
12	Rutin	C_27_H_30_O_16_	610.52	*Ruta graveolens*; *Fagopyrum esculentum*; *Sophora japonica*	Kwon et al. (2005); Mascaraque et al. (2014); Sharma et al. (2021)

Flavanones					
13	Alpinetin	C_16_H_14_O_4_	270.28	*Alpinia japonica*; *Alpinia katsumadae*	He et al. (2016); Lv et al. (2018); Tan and Zheng (2018)
14	Didymin	C_28_H_34_O_14_	594.56	*Clinopodium chinense*; *Citrus medica*; *Citrus limon*	Lv et al. (2021)
15	Eriodictyol	C_15_H_12_O_6_	288.25	*Scutellaria barbata*; *Citrus limon*; *Arachis hypogaea*	Hu L. H et al. (2021); Wang R et al. (2021)
16	Hesperetin	C_16_H_14_O_6_	302.28	*Citrus sinensis*; *Citrus reticulata*; *Citrus limon*	Polat and Karaboga (2019); Polat et al. (2018); Zhang J. X et al. (2020)
17	Hesperidin	C_28_H_34_O_15_	610.56	*Citrus sinensis*; *Citrus reticulata*; *Citrus limon*	Shafik et al. (2019); Xu et al. (2009)
18	Liquiritigenin	C_15_H_12_O_4_	256.25	*Glycyrrhiza uralensis*; *Glycyrrhiza inflata*; *Glycyrrhiza glabra*	Min et al. (2015)
19	Naringenin	C_15_H_12_O_5_	272.25	*Citrus aurantium*; *Citrus paradisi*; *Citrus reticulata*; *Scutellaria barbata*	Al-Rejaie et al. (2013); Dou et al. (2013)
20	Naringin	C_27_H_32_O_14_	580.53	*Citrus aurantium*; *Citrus paradisi*; *Citrus reticulata*; *Scutellaria barbata*	Cao et al. (2018); Cao et al. (2021); Dong et al. (2021); Hambardikar and Mandlik (2022); Kumar et al. (2014)
21	Pinocembrin	C_15_H_12_O_4_	256.25	*Alpinia katsumadae*; *Pinus wallichiana*; *Zingiber officinale*	Hu et al. (2019); Yue et al. (2020)

**TABLE 2:** Molecular mechanisms of natural flavonols and flavanones against ulcerative colitis.

Names	Model	Effective dosage	Mechanism of action	References
Flavonols				
Astragalin	DSS evoked UC in C57BL/6J mice	2, 5, 50, 75 and 100 mg/kg in murines	Up: Mucin-2; occludin; ZO-1; *Verrucomicrobia*; *Ruminococcaceae;* Lachnospiraceae; *Butyricicoccus*; *Ruminococcaceae* *_NK4A214_group*; *Ruminococcaceae* *_UCG-009*; *Ruminiclostridium*; *Oscillibacter*; *Ruminiclostridium_9*	Han et al. (2021); Peng et al. (2020)
	TNF-α activated HCT-116 or HT-29 cells	10 and 50 μM in cells	Down: MCP-1; TNF-α; IL-1β; IL-6; IFN-γ; COX-2; MPO; LPS; TLR4; p-IκBα/IκBα; p-p65/p65; p-IKKα/β/IKKα; p-IKKα/β/IKKβ; *Proteobacteria; Prevotellaceae, Erysipelotrichaceae, Bacteroidaceae, Peptostreptococcaceae, norank_o__Rhodospirillales*; *Ruminococcus_1*; *Bacteroides*; *Escherichia-Shigella*	
Fisetin	DSS evoked UC in Balb/C mice	10 mg/kg in murines	Up: GSH; IκBα	Sahu et al. (2016)
	LPS activated peritoneal macrophages	10 and 20 μM in cells	Down: Nitrites; TNF-α; IL-1β; IL-6; COX-2; iNOS; MPO; MDA; NF-κB; NF-κB (nuclear); p-NF-κB; p-IκBα/IκBα; p-p38/p38; p-ERK/ERK; p-Akt/Akt	
Galangin	DSS evoked UC in Swiss albino mice or ICR mice	15, 20 and 40 mg/kg	Up: GSH; p-AMPK; ATG5; ATG7; ATG12; LC3B; *Firmicutes*/*Bacteroidetes*; *Lactobacillus* spp.; *Butyricimonas* spp.; *Mucispirillum* spp.; acetic acid; propionic acid; butyric acid; total SCFAs	Gerges et al. (2020); Xuan et al. (2020)
			Down: LDH; TLR4; p65; TNF-α; IL-β; IL-6; MPO; HMGB1; MDA	
Hyperoside	DSS evoked UC in C57BL/6 mice	3, 10, and 30 mg/kg in murines	Up: Mucin2; TJP1; occludin; ZO-1; claudin-5; Tregs; Foxp3; IL-10; TGF-β; CD36; LPL; PPARγ	Cheng et al. (2021)
	Caco-2 cells	3, 10, and 30 μM in cells	Down: TNF-α; IL-1β; IL-6; Th17 cells; Th17 cells/Tregs; RORγt; IL-17; IL-22; IL-23; MKRN1	
Icariin	DSS evoked UC in C57BL/6 mice	3, 10, 30, 60, 90 mg/kg in murines	Up: *Lachnospiraceae; Akkermansia*; *Lactobacillus*	Tao et al. (2013); Wang et al. (2016); Zhang et al. (2021)
	TNBS evoked UC in SD rats	0.3, 1, 3 and 10 μM in cells	Down: TNF-α; IL-1β; IL-2; IL-6; IFN-γ; IL-17A; IL-17F; PGE_2_; NO; STAT3; p-STAT1; p-STAT3; p65; p-p65; CD25; CD69; iNOS; COX-2; *Tenericute*; *Deferribacteres*; *Helicobacteriaceae; Bacteroides*; *Turicibacter*	
	IFN-γ or IL-6 activated Naive T cells			
Kaempferol	DSS evoked UC in C57BL/6 mice	50 mg/kg	Up: TFF3; ZO-1; occludin; claudin-1; *Firmicutes*/*Bacteroidetes*; D-fructose 2,6-bisphosphate; D-xylose; galactitol; lactose; N-acetyl-5-hydroxytryptamin	Park et al. (2012); Qu et al. (2021)
			Down: MPO; LTB_4_; NO; COX-2; iNOS; PGE_2_; TNF-α; IL-1β; IL-6; LPS; TLR4; MyD88; p-p65; NLRP3; *Proteobacteria*; *Gammaproteobacteria*; *Enterobacteriales*; Enterobacteriaceae; *Escherichia_Shigella*	
Morin	TNBS evoked UC in Wistar rats	5, 10, 25, 100 and 200 mg/kg	Up: GSH	Gálvez et al. (2001); Ocete et al. (1998)
			Down: MPO; LTB_4_; MDA; IL-1β; NOS	
Morusin	TNBS evoked UC in Wistar rats	12.5, 25, and 50 mg/kg	Up: Pro-MMP2/MMP2	Vochyanova et al. (2017)
			Down: TGF-β1; MMP2; Pro-MMP2; MMP9; IL-1β	
Myricitrin	DSS evoked UC in CD1 mice	1, 3, and 10 mg/kg	Down: p-Akt; p-p38; p-JNK; p-ERK; NF-κB p65; PKC-ε; NOS2; COX-2; IL-6; CXCL1/KC; TNF-α	Schwanke et al. (2013)
Quercetin	Transfer of CD4^+^CD25^−^CD62L^+^ T cells to Rag1^−/−^ mice	10, 30 mg/kg 100, 500, 1000, 1500 ppm in murines	Up: Goblet cells; CD11b^+^ F4/80^+^ cells; CD11b; CD11b^+^ macrophages; Ym1; Fizz1; Arg1; TGF-β1; IL-10; GCLC; GCLM; HO-1; SRXN1; NQO1; Nrf2; GSH; SOD; *Firmicutes*; *Bacteroides*; *Bifidobacterium*; *Lactobacillus*; *Clostridia*; MRP1; GR; NOX1; NOX2; ERK1/2	Dong et al. (2020a); Dong et al. (2020b); Ju et al. (2018); Lin et al. (2019)
	DSS evoked UC in C57BL/6J mice	6.25, 12.5, and 25 μM in cells	Down: TNF-α; IL-6; IL-12; IL-17A; IL-17; IL-23; IFN-γ; CD4^+^ T cells; CD25; CD69; CD40L; 4-1BB; CD80; CD86; CD40; 4-1BBL; LPS; iNOS; 16S rDNA; ROS; CAT; AQP3; apoptosis; NR3C1; TP63; EHF; STAT3; estrogen receptor; TGFB1; *Proteobacteria*; *Actinobacteria*; segmented filamentous bacteria; *Escherichia coli*; *Bacteroidetes*; *Verrucomicrobia*; *Fusobacterium*; *Enterococcus*	
	*C. rodentium* evoked UC in C57BL/6 mice			
	H_2_O_2_ activated Caco2 cells			
Isoquercitrin	DSS evoked UC in Wistar rats	1, 10 mg/kg	Down: COX-2; iNOS	Cibicek et al. (2016)
Rutin	DSS evoked UC in Balb/C mice	25, 50 and 57 mg/kg	Up: Mucin-2; mucin-3; CYP11B1; CD4^+^ CD25^+^ cells; PCNA; Bcl-2	Kwon et al. (2005); Mascaraque et al. (2014); Sharma et al. (2021)
	Transfer of CD4^+^CD62L+ T cells to Rag1^−/−^mice		Down: TNF-α; IL-1β; IL-6; IL-17; IFN-γ; REG3γ; MMP3; CXCL1; S100A8; GM-CSF; iNOS; COX-2; MPO; AP; p-STAT4; p-IκBα; HO-1; ICAM; IgM; IgA; TNF-α/IL-10; IL-1β/IL-10; IL-6/IL-10; IFN-γ/IL-10; IL-5/IL-10; p-p38/p38; p-MK2/MK2; p-NF-κB/NF-κB; p-Akt/Akt; CYP11A1; CD3^+^ CD4^+^ cells; CD3^+^ CD8^+^ cells; lymphocytes; monocytes; neutrophil; Bax	
**Flavanones**				
Alpinetin	DSS evoked UC in BALB/c or C57BL/6 mice	7.5, 15, 25, 30, 50 and 100 mg/kg in murines	Up: Occludin; ZO-1; SOD; Nrf2; HO-1; IL-10; Foxp3; CD4^+^CD25^+^Foxp3^+^ T cells; AhR; CYP1A1; miR-302	He et al. (2016); Lv et al. (2018); Tan and Zheng (2018)
	LPS activated THP-1 cells	50, 100 and 200 μg/ml in cells	Down: MPO; TNF-α; IL-1β; p-IκB; p-p65; TLR4; NLRP3; ASC; caspase-1; claudin-2; MDA; IL-17; RORγt; CD4^+^IL-17^+^ T cells; DNMT-1	
Didymin	DSS evoked UC in C57BL/6 mice	1,2 and 4 mg/kg in murines	Up: F4/80^+^ CD206^+^ cells; Arg1; Retnla; Chil3; IL-10	Lv et al. (2021)
	LPS + IFN-γ activated BMDMs	1, 3, 10 and 30 μM in cells	Down: MPO; CD11b^+^ Ly6G^+^ cells; F4/80^+^ NOS2^+^ cells; TNF; IL-1β; IL-6; NOS2	
Eriodictyol	TNBS evoked UC in Wistar rats	5, 20, 40, and 50 mg/kg	Up: IL-10; SOD; CAT; GSH-Px; ZO-1; occludin; IκBα; Bcl-2; Ptc; Shh; Smo; Gli1	Hu Y et al. (2021); Wang Y. F et al. (2021)
	DSS evoked UC in C57BL/6 mice		Down: MPO; MDA; TNF-α; IL-1β; IL-2; IL-6; IL-12; IL-17; IL-23; TLR4; p-IκBα; p-p65/p65; Bax; cleaved caspase 3	
Hesperetin	TNBS evoked UC in Wistar rats	20 or 100 mg/kg in murines	Up: ZO-1; occludin; mucin-2; IL-10	Polat and Karaboga (2019); Polat et al. (2018); Zhang X. L et al. (2020)
	DSS evoked UC in C57BL/6 mice	100 μM in cells	Down: MPO; MDA; TNF-α; IL-1β; IL-6; IL-18; NF-κB; CD45; Cl-caspase-3; Bax; HMGB1; p-RIPK3; p-MLKL	
	LPS activated Caco-2 and RAW264.7 cells			
Hesperidin	DSS evoked UC in BALB/c mice or Wistar rats	10, 40, 50 and 80 mg/kg	Up: PGC-1α; TAC; SOD; Bcl-2	Shafik et al. (2019); Xu et al. (2009)
			Down: MPO; MDA; IL-6; SGPP2; SphK1; MIP-1α; NO; peroxynitrite	
Liquiritigenin	TNBS evoked UC in ICR mice DSS evoked UC in C57BL/6 mice	10, 20 mg/kg	Up: IL-10	Min et al. (2015)
			Down: TNF-α; IL-1β; IL-6; p-IKKβ; p-p65; p-IκBα	
Naringenin	AA evoked UC in Wister albino rats	25, 50 and 100 mg/kg in murines	Up: T-GSH; NPSH; CAT; SOD	Al-Rejaie et al. (2013); Dou et al. (2013)
	TNF-α activated HT-29 cells	1, 10 and 25 μM in cells	Down: TLR4; p-p65; p-IκBα; iNOS; ICAM-1; MCP-1; COX-2; TNF-α; IL-6; IL-1β; TBARS; PGE_2_; NO	
	LPS activated RAW264.7 cells			
Naringin	AA evoked UC in Wistar rats	20, 25, 40, 50, 80, 100 mg/kg in murines	Up: SOD; GSH; CAT; WBC; RBC; Hb; PLT; PPARγ; PPARα; ZO-1; occludin; *Firmicutes*; *Firmicutes*/*Bacteroidetes*	Cao et al. (2018); Cao et al. (2021); Dong et al. (2021); Hambardikar and Mandlik (2022); Kumar et al. (2014)
	DSS or TNBS evoked UC in C57BL/6 mice	10, 20 and 40 μM in cells	Down: LDH; SGPT; SGOT; ALP; MDA; MPO; NO; XO; TNF-α; IL-1β; IL-6; IL-12; IFN-γ; p-p65; p-IκB; p-p38; p-ERK; p-JNK; NLRP3; ASC; caspase-1; Cl-caspase3; iNOS; COX-2; *Bacteroidota*; Proteobacteria; *Campilobacterota*; *Deferribacterota*; *Verrucomicrobiota*	
	TNBS evoked UC in Wistar albino rats			
	LPS activated RAW264.7 or IEC-6 cells			
Pinocembrin	DSS evoked UC in SD rats or C57BL/6 mice	5, 10, 25, 50 and 100 mg/kg in murines	Up: TGF-β; occludin; ZO-1; claudin-1; JAM-A; goblet cells; SCFAs; acetate; propionate; butyrate; *Firmicutes*; *Lactobacillus* spp., *Alloprevotella* spp., *Desulfovibrio* spp	Hu et al. (2019); Yue et al. (2020)
	LPS activated RAW264.7 and Caco-2 cells	37.5, 75 and 150 μM in cells	Down: TNF-α; IL-1β; IL-6; IL-15; TLR4; MyD88; iNOS; COX-2; IFN-γ; p-IκBα/IκBα; p-p65/p65; TLR4/MD2; *Protebacteria*; Enterobacteriaceae; *Escherichia_Shigella*; *Enterococcus*	

### Natural flavonols

Astragalin is an active ingredient of many medicinal plants, such as *Astragalus membranaceus*, *Astragalus sinicus*, and *Thesium chinense*. Modern studies manifested that astragalin has many properties, including anti-inflammation, anti-oxidation, neuroprotection, and cardioprotection (Riaz et al., 2018; Kim et al., 2022). Peng et al. (2020) explored the therapeutical effect and mechanism of astragalin on murine UC evoked by DSS. The evidences displayed that astragalin decreased weight loss and DAI, restrained colonic shortening, and relieved colon tissue injury. Astragalin lowered the levels of inflammatory factors and relevant mRNA (TNF-α, IL-6, and IL-1β), suppressed macrophage and neutrophil infiltration in colonal tissues, alleviated metabolic endotoxaemia, and enhanced intestine mucosa barrier function. Western blotting results showed that astragalin downregulated the NF-κB signal path. Furthermore, astragalin treatment could reverse the changes in the intestinal flora in UC murines, mainly through elevating the quantity of benefic bacteria and reducing the quantity of maleficent bacteria. Thus, astragalin exerted a fine anti-colitis role through microflora/LPS/TLR4/NF-κB-relevant paths in murines. In conclusion, the results demonstrated the anti-inflammatory role and mechanism of astragalin and offered a significant basis for probing the mechanisms of natural flavonols associated with suppressing inflammation-driven disorders. Han et al. (2021) investigated the anti-inflammation role of astragalin *via* blocking NF-κB signal path in colon epithelia cells and UC murines. The results indicated that astragalin exerted an anti-inflammatory role by the suppression of NF-κB path. Astragalin may be an exciting therapeutical medication for the treatment of UC.

Fisetin is a flavonol that is, extensively existed in edible or pharmaceutical plants, such as *Fragaria ananassa*, and *Toxicodendron sylvestre*. It has been found to possess anti-inflammatory, anticancer, antioxidative, and neuroprotective actions (Yousefzadeh et al., 2018; Ul Hassan et al., 2022). Sahu et al. (2016) investigated the role and mechanism of fisetin on colitis in DSS-elicited mice. Fisetin administration markedly lowered the seriousness of UC and relieved the clinical symptoms. Furthermore, fisetin reduced the MPO vitality, the levels of proinflammatory mediators, and the expressions of COX-2 and iNOS in the colon. Further researches proved that fisetin inhibited the excitation of p65 through suppressing IκBα phosphorylation and p65-DNA binding activity and decreased the phosphorylation of Akt and the p38 in the colonal samples of DSS-disposed murines. DSS-caused reduction in GSH and rise in MDA contents were notably recovered by fisetin. Besides, the result of *in vitro* experiment indicated that fisetin markedly decreased the production of proinflammatory mediators and restrained the degradation and phosphorylation of IκBα with succedent nuclear transsituation of p65 in murine primary peritoneal macrophage excited by LPS. The proofs suggested that fisetin exerted an anti-inflammatory role by suppressing Akt, MAPK and NF-κB signal in the colons of DSS-treated murines. Therefore, fisetin could be a potential candidate drug in the therapy of colitis.

Galangin is the dietary and active constituent of *Alpinia officinarum*, which has various pharmacological activities like anti-cancer, anti-inflammation, anti-oxidation, and liver protection (Rampogu et al., 2021; Tuli et al., 2022). Xuan et al. (2020) investigated the function and mechanism of galangin against DSS elicited mice colitis. The proofs indicated that galangin caused a remarkable improvement on UC symptoms, involving a reduced DAI, suppression of the colonic length shortening, and relief of the pathologic variations appearing in the colons. Colonal pro-inflammatory factors, involving TNF-α, IL-1β and IL-6, and MPO activity were reduced after galangin administration in comparation with the model group. Furthermore, galangin notably elevated ATGs level and facilitated autophagosome formation in the colon. Galangin elevated the diversity of the intestinal microflora along with elevated levels of SCFAs. These variations were associated with the roles of galangin in regulating certain specific bacterial populations, involving the restoration of *Lactobacillus* spp. and elevated *Butyricimonas* spp. Gerges et al. (2020) also investigated the effect and potential mechanism of galangin treating UC induced by DSS in mice. The results proved that galangin notably alleviated DSS-treated histopathologic changes and tissue damage, down-regulated TLR4 expression, restrained NF-κB p65 excitation, reduced inflammatory factor levels, and indicated antioxidative roles. In conclusion, galangin may be a viable treatment choice for colitis.

Hyperoside, a natural flavanol glycoside, mainly comes from plants of the genera *Hypericum* and Crataegus and also exists in various vegetables and fruits. It was reported to possess diversified activities involving anti-inflammation, anti-bacteria, anti-cancer, anti-oxidation, and immunoregulation (Wang et al., 2022; Xu et al., 2022). In a work by Cheng et al. (2021), they investigated the function and mechanism of hyperoside against colitis in DSS-elicited UC mice. Experimental evidences displayed that hyperoside decreased the pathologic score, maintained tissue integrality, inhibited colon inflammation, and balanced the Treg response. Hyperoside was proven to suppress the levels of E3 ubiquitin ligase and MKRN1, which in turn facilitated the ubiquitination and proteasomal degradation of PPARγ, a necessary modulator of Th17 and Treg differentiation. Therefore, hyperoside administration improved PPARγ signal and so facilitated Treg differentiation while inhibiting Th17 cell development. In conclusion, hyperoside played a role *via* the MKRN1/PPARγ axis to regulate the Th17/Treg axis and so provides protection on UC. These discoveries expand our knowledge on hyperoside effect and may offer a potential therapeutical target for colitis.

Icariin is a primary active constituent of *Epimedium brevicomu*, *Epimedium sagittatum*, *Epimedium pubescens*, and *Epimedium koreanum*. Pharmacological investigations proved that icariin has anti-atherosclerotic, anti-cancerous, anti-inflammatory, and neuroprotective activities (He et al., 2020; Zeng et al., 2022). Tao et al. (2013) investigated the protection and mechanism of icariin against UC induced by DSS in mice. Icariin treatment notably relieved the disease progression and the pathologic variations of UC. Additionally, icariin suppressed the generation of pro-inflammatory factors and expressions of p-p65, p-STAT1 and p-STAT3 in colonic samples. Further research indicated that icariin concentration-dependently restrained T lymphocyte activation and proliferation as well as pro-inflammatory factor levels in activated T cells. Furthermore, icariin suppressed the phosphorylations of STAT1 and STAT3 in CD4^+^ T cells, which were separately the significant transcription factors for Th1 and Th17. Collectively, the results certified that icariin is a promising therapeutical drug for colitis. In the research of Wang et al. (2016), the protective properties of alginate-chitosan microspheres loaded with icariin were explored in rats with colon mucosa injury caused by TNBS/ethanol treatment. The results showed that the targeted microspheres loaded with icariin could lower the colonic mucosal damage indicator through reducing the production and gene expression of inflammatory mediators. The microspheres cross-linked by glutaraldehyde elevated the retention of medications in the colons and guarded against their reduction in the upper and middle parts of the alimentary system. Thus, the targeted microspheres loaded with icariin may be a novel therapy for UC. Zhang et al. (2021) also explored the role and mechanism of icariin on the enteric microbiota of UC mice. The proofs indicatd that icariin could improve the gut microflora abundance and structure of DSS-treated UC murine, and suppress colon injury and inflammatory reaction. In brief, icariin could be a promising strategy for treating UC.

Kaempferol, a flavonol aglycone found in various fruits and vegetables, has numerous therapeutical effects, including anti-oxidation, anti-tumor, and anti-inflammation (dos Santos et al., 2021; Imran et al., 2019). In the research of Park et al. (2012), they determined whether kaempferol relieved the inflammatory reactions of DSS-induced UC in murines. Plasma NO and PGE_2_ levels and colon mucosal MPO activity were markedly reduced after 0.3% kaempferol pre- and post-treatment. The plasma LTB_4_ level was observably reduced in all murines administrated with kaempferol. TFF3, a marker for goblet cell function, was elevated in kaempferol pre-treatment mice. Collectively, the results indicated that kaempferol is a fine anti-inflammatory drug which shields colon mucosa from DSS-treated colitis. In another work, Qu et al. (2021) also evaluated the protection and mechanism of kaempferol in DSS-elicited colitis murines. The proofs revealed that kaempferol exerted an immunoregulatory effect in colitis murines through regulating the enteric flora and various metabolites, thus inhibiting the LPS-induced TLR4-NF-κB signal pathway. In conclusion, this work offers a new view that foods rich in kaempferol may possess a healthy benefit for the prevention of colitis.

Morin is a dietary flavonol mainly acquired from the plants of Moraceae family. It has been proven that morin possesses multiple pharmacologic actions, including anti-oxidation, anti-inflammation, anti-cancer, anti-microbe, and anti-diabetes (Kataria et al., 2018; Mottaghi and Abbaszadeh, 2021). In study by Ocete et al. (1998), they probed the anti-inflammatory role of morin in TNBS-treated rat colitis. The results showed that morin increased GSH level and decreased MPO activity, LTB_4_ synthesis and MDA level, indicating that the mechanism of morin against colitis is associated with the suppression of colon LTB_4_ synthesis and antioxidant properties. In another research, Gálvez et al. (2001) assessed the anti-inflammatory role of morin in TNBS-evoked rat colitis. The results indicated that morin facilitated tissue recovery and reduced MPO vitality. The intestine anti-inflammatory role of morin was accompanied by a remarkable decrease in colon LTB_4_ and IL-1β levels, enhancement in colon oxidant stress and suppression of colon NO synthase activity. In conclusion, morin exerted a beneficial anti-inflammatory role on UC by the down-modulation of certain mediators associated with the gut inflammatory reaction, covering free radicals, cell factors, LTB_4_ and NO. Therefore, morin is expected to be an excellent candidate medicine for the remedy of colitis.

Morusin, a prenylated dietary flavonol found in the root barks of *Morus alba*, has been reported to possess multiple properties, including anti-oxidation, anti-tumor, anti-inflammation, and anti-allergy (Xue et al., 2018; Choi et al., 2020). Vochyanova et al. (2017) investigated the effect and mechanism of morusin against UC elicited by TNBS in Wistar rats. The results showed that the tissue injury scores were observably decreased with increasing dosage of morusin, but the therapeutic effect was not indicated at the highest dosage. Morusin had a therapeutic effect at a dosage of 12.5 mg/kg that was comparable to sulfasalazine’s (50 mg/kg) impact. This was related to a prominent decrease in TGF-β1, MMP2 and MMP9 levels, and mild decrease in IL-1β. These proofs suggested the therapeutical potential of morusin for the management of colitis.

Myricitrin, a usual dietary flavonol, is widely existed in *Myrica cerifera*, *Eugenia uniflora*, and *Ampelopsis grossedentata*. Multiple functions, including anti-oxidation, anti-inflammation, and anti-nociception have been attributed to it (Domitrovic et al., 2015; Zhang X. L. et al., 2020). In the work by Schwanke et al. (2013), they explored the protective action of myricitrin in DSS-induced UC as a potential treatment option for UC. The results indicated that myricitrin exerted a consistent anti-inflammatory role in murine UC by suppressing Akt/PI3K-dependent phosphorylation. Therefore, the phosphorylation of MAPK p38, ERK1/2, JNK and NF-κB was decreased, which restrained a rise in the cytokines/chemokines levels. Overall, the results revealed that the anti-inflammatory action of myricitrin in DSS-treated UC mice was tightly related to its capacity to restrain the excitation of upriver kinases including PI3K-dependent Akt, MAPK and NF-κB.

Quercetin, a dietary flavonol widely existed in many plants involving *Styphnolobium japonicum*, *Fagopyrum esculentum*, and *Allium cepa*. It has been proved to possess antidiabetic, antidepressive, anti-inflammatory, and antioxidative activities (Li et al., 2016; Grewal et al., 2021). Lin et al. (2019) explored the action and mechanism of quercetin against UC in *Citrobacter rodentium*-elicited colitis mice. The proofs indicated that quercetin relieved the symptoms of colitis, restrained the generation of pro-inflammatory factors, such as IL-17, TNF-α and IL-6, and elevated IL-10 level in the colonal tissues. Additionally, quercetin increased the populations of *Bacteroides*, *Bifidobacterium*, *Lactobacillus* and *Clostridia*, and observably decreased those of *Fusobacterium* and *Enterococcus*. Therefore, quercetin exerted its anti-colitis role possibly by suppressing pro-inflammatory factors and regulating intestinal microflora. In the research from Ju et al. (2018), they found that quercetin could alleviate experimental UC partly through regulating the anti-inflammatory roles and anti-bacterial activity of macrophages by a HO-1-dependent path. Dong et al. (2020a) demonstrated that quercetin could ameliorate the symptoms of UC in mice treated with DSS and elevate intracellular GSH to remove overproduced ROS stimulated by H_2_O_2_ through up-regulating GCLC and GR, thus improving the proliferation and apoptosis conditions in Caco2 cells. In another study, they also found that quercetin relieved DSS-treated UC probably through reinforcing intestine integrality, liver antioxidative competence, and regulation of certain critical genes associated with the progress of UC, such as the ERK1/2-FKBP path, RXR-STAT3 path (Dong et al., 2020b). Altogether, quercetin may be a potential drug for colitis remedy.

Isoquercitrin is a natural flavonol widely existed in fruits, vegetables, and medicinal plants. Modern studies indicated that isoquercitrin has various activities, including anti-oxidation, anti-inflammation, anti-tumor, and anti-allergy (Valentova et al., 2014; Xie et al., 2016). In the work by Cibicek et al. (2016), they examined whether isoquercitrin effectively and dosage-dependently relieved DSS induced UC rats. The findings demonstrated that isoquercitrin dosage-dependently suppressed colonic shortening and decreased the expressions of COX-2 and iNOS in the descendent colon. Whereas, when diverse sites of the colon were evaluated histomorphometrically, the results could not completely support the protecting action of isoquercitrin. Tissues healing trend observed in the descendent colons were not obvious in the rectum, where histologic injury was most sevious. Together, the effect of isoquercitrin against UC might depend on dosage, the severity of tissue injury, and the site of action.

Rutin is a dietary flavonol broadly found in *Ruta graveolens* and *Fagopyrum esculentum*. Rutin has been found to have antioxidative, anti-inflammatory, antidiabetic, and neuroprotective activities (Ganeshpurkar and Saluja, 2017; Gullon et al., 2017). In the experiment by Sharma et al. (2021), they detected the influence and latent mechanism of rutin relieving UC in DSS-treated colitis mice. The analysis of signal paths proved the strong excitation of PI3K/Akt/GSK3β/MAPKs/NF-κB and p38/MK2 in UC mice, which was effectively relieved after rutin administration. *In silico* researches testified the targeting selectivity of rutin to these paths. Rutin observably ameliorated the DAI scoring, colonic length, goblet cell loss and maintained colonic epithelia integrality in colitis murines. Rutin administration significantly reduced oxidative and inflammatory indicator expressions like HO-1, iNOS, IgM, IgE, ICAM-1 and Th1/IL-10 cytokines ratios. Moreover, rutin proved its effect in keeping epithelia homeostasis and integrality through enhancing IEC proliferation, alleviating apoptosis and regulating the mRNA expression of TJ proteins and mucus proteins. Additionally, Treg amplification showed that rutin exhibited immune modulatory role and suppressed inflammatory exacerbation activated by adaptive immune reactions. These findings indicated that the regulation of p38/MK2 and PI3K/Akt/GSK3β/NF-κB paths by rutin represented a new therapeutical strategy for UC, which is helpful to restrain disregulated intestine integrality, cytokine ratio and splenic Tregs. Kwon et al. (2005) probed the protection and mechanism of rutin against UC in DSS-treated murines. The results showed that rutin notably mitigated the production of pro-inflammatory mediators IL-1β, IL-6, granulocyte macrophage-colony stimulating factor (GM-CSF) and iNOS, thus alleviating DSS-treated UC in murines. In the experiment by Mascaraque et al. (2014), they probed the anti-inflammatory role of rutin against UC in the CD4^+^ CD62L+ T cell transfer model of UC. Experimental proofs displayed that rutin exerted an anti-inflammatory role on T lymphocyte dependent colitis through inhibiting inflammatory cytokines. In conclusion, rutin may be an effective medication for the management of UC.

### Natural flavanones

Alpinetin, the major active constituent in *Alpinia katsumadai* and *Alpinia japonica*, was proved to possess anticancer, antiphlogistic, and hepatoprotective effects (Wikan et al., 2022; Zhao et al., 2022). In the experiment of He et al. (2016), they investigated the anti-inflammatory role and mechanism of alpinetin on DSS-treated UC in murines. The results indicated that alpinetin observably alleviated diarrhoea, colon shortening, histologic damage, MPO vitality and the expressions of TNF-α and IL-1β production in murines. *In vitro*, alpinetin notably suppressed LPS-evoked TNF-α and IL-1β production, and TLR4 mediated NF-κB and NLRP3 inflammasome excitation. In the experiment from Tan and Zheng (2018), they investigated the role of alpinetin on gut epithelia TJs, oxidant stress and Nrf2/HO-1 signal path in DSS-treated colitis in murines. The results showed that alpinetin elevated DAI, colon shortening, histologic score, and MPO activity in comparation with the DSS group. The levels of occludin and ZO-1 were increased by alpinetin, but the level of claudin-2 was decreased. Furthermore, alpinetin suppressed MDA content, and elevated SOD content. Nrf2/HO-1 signal paths were also discovered to be excited. Overall, the results suggested that the integrality and penetrability of the gut epithelia barrier are protected by alpenetin through modulating the levels of TJ proteins, suppressing oxidant stress and stimulating Nrf2/HO-1 signal path. In another experiment, Lv et al. (2018) estimated the role of alpinetin on DSS-treated colitis and elucidated the latent mechanism. Experimental evidence displayed that alpinetin mitigated UC in mice by stimulating AhR, modulating miR-302/DNMT-1/CREB signals, thus contributing to Treg differentiation. Collectively, these studies indicated that alpinetin had protective roles on DSS-treated UC and may be a potential therapeutical drug for UC.

Didymin is a bioactive dietary flavonoid glycoside firstly discovered in citrus fruits. Emerging studies proved the promising therapeutical application of dietary didymin against tumor, nerve disorders, hepatic disorders, and cardiovascular disorders (Singhal et al., 2017; Yao et al., 2018). In the research from Lv et al. (2021), they firstly proved that didymin markedly relieved the clinic symptoms of colitis in murines. Mechanism studies indicated that didymin converted pro-inflammatory M1-like to anti-inflammatory M2-like macrophage phenotype, but did not change the polarization of M2-like macrophages. Metabolic tracking research found that didymin enhanced fatty-acid oxidation rather than glycolysis *via* the induction of Hadhb expression. Furthermore, *in vivo* researches proved that rise of Hadhb expression led to the conversion of M1-toward M2-like macrophages and ultimately relieved UC. In conclusion, the results highlighted the capacity of macrophage paradigm in colitis inflammation and put forward the stage for regarding didymin as a metabolic modulator in reprogramming macrophage polarization, which might be a prospective therapeutical method for treating inflammation-related diseases.

Eriodictyol, a natural flavanone existed in citrus fruits and vegetables, has been testified to have diversified properties comprising anti-inflammation, anti-apoptosis, and anti-oxidation (Deng et al., 2020; Islam, 2020). Wang R. et al. (2021) probed the protecting role and mechanism of eriodictyol in colitis. The data displayed that, compared with the DSS group, the eriodictyol administration group notably reduced DAI scores, colon shortening, and histologic scores. Eriodictyol also decreased inflammatory responses, oxidant stress, and apoptosis in the colons. Moreover, eriodictyol elevated the levels of the TJ proteins ZO-1 and occludin. Mechanistically, eriodictyol up-modulated the Shh signal path. Whereas, Cyc-mediated suppression of the Shh path partially abolished the action of eriodictyol. Collectively, the result showed that eriodictyol attenuated DSS-treated UC *via* stimulating the Shh path. Hu L. H. et al. (2021) also investigated the function and mechanism of eriodictyol in restraining colitis. The results showed that eriodictyol relieved TNBS-treated intestine tissue damage in rats. It was discovered that eriodictyol decreased MPO expression and modulated the cytokine parameters in TNBS-treated intestine tissues of murines. The levels of TNF-α, IL-1β, IL-6, IL-10, IL-2, and IL-12 were also influenced by eriodictyol administration. Eriodictyol also influenced SOD, catalase (CAT), GSH-Px, and MDA levels in UC rats. Furthermore, eriodictyol modulated TNBS-evoked TLR4/NF-κB path excitation, thus suppressing the progress of colitis. In brief, the evidence suggests that eriodictyol may be a viable therapeutical drug for colitis.

Hesperetin is a dietary flavanone widely found in citrus plants, including *Citrus limon*, *Citrus sinensis*, and *Citrus reticulata*. It has been certified to have diversified activities involving anti-oxidation, anti-inflammation, anti-tumor, and anti-hypertension (Muhammad et al., 2019; Khan et al., 2020). Among the experiment of Zhang J. X. et al. (2020), they investigated the protective action and mechanism of hesperetin on DSS-evoked UC. Experimental data showed that hesperetin signally mitigated the syndromes of DSS-induced UC, enhanced the expression of ZO-1, occludin and mucin-2, and decreased TNF-α, IL-1β, IL-18, HMGB1 and IL-6. Significantly, IHC and western blot assays proved that hesperetin suppressed the expression of RIPK3 and MLKL, the two critical proteins of necroptosis path, and devitalized RIPK3/MLKL necroptosis signal. Simultaneously, in the cell-coculture system between Caco-2 and RAW264.7 cells, hesperetin observably restrained the decline of transepithelial electrical resistance values, but necroptosis inducer could notably affect the role of hesperetin. Besides, hesperetin relieved the LPS-caused increase in 4-kDa FD4 penetrability, but HS-173 could impair the protecting role of hesperetin. In the meantime, HS-173 decreased the variations in the expression of phosphorylated RIPK3, phosphorylated MLKL, ZO-1, occludin and mucin-2 as well as TNF-α, IL-1β. Therefore, hesperetin could ameliorate DSS-evoked UC through preserving the epithelia barrier by preventing the intestine epithelia necroptosis. In two other studies, Polat et al. probed the mechanism of hesperetin against UC in TNBS-evoked colitis rats (Polat et al., 2018; Polat and Karaboga, 2019). The results demonstrated that hesperetin could protect against UC through its anti-inflammatory, anti-oxidative, and anti-apoptotic roles. Taken together, hesperetin may be a viable therapeutical drug for the therapy of UC.

Hesperidin is dietary flavonoid glycoside abundant in citrus plants and has been testified to have diversified anti-inflammation, anti-oxidation, anti-virus, and analgesia (Li and Schluesener, 2017; Hajialyani et al., 2019). Xu et al. (2009) explored the function and mechanism of hesperidin on DSS-elicited experimental UC in murines. Experimental proofs displayed that hesperidin prominently reduced the DAI score, MPO vitality, MDA content, and IL-6 level in serum but exerted no significant role on the level of IL-4 in serum. The research from Shafik et al. (2019) also offered some opinions into hesperidin regulatory roles on colitis pathogeny, which may be owed to suppressing the SphK1-S1P signal path, therefore restraining the downriver inflammatory and apoptotic cascades characterized by depressed MIP-1α and advancement of bcl2 expression. Hesperidin also improved mitochondria biosynthesis *via* elevating PGC1-α expression, and recovered redox potential characterized by remarkable declines of NO and peroxynitrite, elevating total antioxidant capacity and stimulating SOD. In conclusion, hesperidin may be a novel therapeutical strategy for colitis.

Liquiritigenin, a major flavanone extracted from *Glycyrrhiza uralensis* and *Glycyrrhiza inflata*, possesses diversified influences comprising cytoprotection, anti-allergen, anti-inflammation, and immunoregulation (Jo et al., 2016; Ramalingam et al., 2018). In the experiment from Min et al. (2015), they probed the anti-inflammatory role and mechanism of liquiritigenin for TNBS-treated UC in murines. The evidences displayed that liquiritigenin significantly increased body weight, inhibited colonic shortening, alleviated histologic injuries and decreased MPO vitality in the colon tissues. Moreover, liquiritigenin markedly lowered the levels of TNF-α, IL-1β, IL-6 and elevated IL-10 level. Besides, liquiritigenin observably lowered TNBS-evoked phosphorylation of IKKβ, p65 and IκB-α. In conclusion, liquiritigenin has the potentiality to be a novel candidate drug of UC therapy.

Naringenin is a dietary flavanone abundantly found in *Citrus reticulata*, *Citrus aurantium*, and *Citrus paradisi*. It has been certified to possess diversified activities covering anti-tumor, anti-oxidation, and anti-inflammation (Alam et al., 2014; Salehi et al., 2019). In the experiment from Dou et al. (2013), they explored the influence and mechanism of naringenin in DSS-evoked mice colitis. Naringenin observably decreased the seriousness of UC and led to down-modulation of pro-inflammatory factors (iNOS, ICAM-1, MCP-1, Cox2, TNF-α and IL-6 mRNA) in the mucosae of colons. The decline of proinflammatory factors (especifically TNF-α and IL-6) was related to a reduction in mucosa TLR4 mRNA and protein. Phospho-NF-κB p65 protein was markedly reduced, which was associated with a similar decline in phospho-IκBα protein. In accordance with the *in vivo* evidence, naringenin administration inhibited lipopolysaccharide-activated nuclear transsituation of NF-κB p65 in murine macrophage RAW264.7 cells. Moreover, *in vitro* NF-κB reporter experiments conducted on human colon HT-29 cells exposed to naringenin showed a prominent suppression of TNF-α-caused NF-κB luciferase expression. Therefore, the results indicated that targeted suppression of the TLR4/NF-κB signal path may be a significant mechanism for naringenin treating UC. In another work, Al-Rejaie et al. (2013) investigated the effect and mechanism of naringenin on acetic acid-evoked UC in rats. Experimental evidences proved that naringenin protected the acetic acid-induced colitis through restraining inflammatory and oxidant indicators. Altogether, naringenin may be a viable candidate drug for colitis.

Naringin is a dietary flavonoid glycoside and found to present strong anti-inflammatory and antioxidant functions. Some investigations suggest that naringin supplementation is salutary for the therapy of gastrointestinal disorders (Alam et al., 2014; Ahmed et al., 2019). In the experiment of Kumar et al. (2014), they valuated the function of naringin on experimentally evoked colitis in rats. The results proposed that naringin possesses an anti-inflammatory, anti-oxidative, and anti-apoptotic possible role at colorectal sites as it regulates the production and expression of oxidation indicators, including MDA, MPO, NO, and XO, thus decreasing DNA injury. Among the work of Cao et al. (2018), they explored the potential mechanism of naringin against DSS-evoked colitis. The results displayed that naringin markedly mitigated DSS-evoked UC outcomes, and the anti-colitis function of naringin was related to the excitation of PPARγ. Moreover, naringin observably inhibited DSS-evoked NLRP3 inflammasome excitation and modulated ZO-1 expression. Dong et al. (2021) investigated the role and mechanism of naringin on UC in DSS or TNBS evoked colitis murines, and found that PPARγ was the major target of naringin based on functional tests both *in vivo* and *in vitro*. Cao et al. (2021) also explored the role and mechanism of naringin on DSS-evoked UC in murines. It was discovered that naringin could markedly relieve the pathogenic symptoms of colitis by suppressing inflammatory reactions and modulating gut microflora. In the research from Hambardikar and Mandlik (2022), they probed the role and mechanism of naringin on TNBS-treated UC in rats. The evidences testified that naringin alleviated the pathological variations of colitis by recovering colon injury and decreasing inflammatory reaction in the colonic tissues. In conclusion, naringin may be regarded as an effective candidate for colitis.

Pinocembrin is a main flavanone separated from various plants involving *Alpinia katsumadae*, *Pinus wallichiana*, and *Zingiber officinale*. Studies showed that pinocembrin has diversified bioactivities comprising anti-microbe, anti-inflammation, and anti-oxidation (Lan et al., 2016; Shen et al., 2019). In the study of Hu et al. (2019), a DSS-evoked UC rat model was adopted to probe the protective effect of pinocembrin on macroscopic clinical signs, inflammatory responses, intestine epithelia barrier function, and intestinal flora homeostasis. However, DSS-treated murines exhibited serious UC clinic signs and histologic variations (colon pathologic injuries and intestine goblet cells reduction), administration of pinocembrin for 7 days relieved these symptoms. Pinocembrin administration also restrained the pro-inflammatory gene expressions and elevated TJ functions of colon epithelia cells. Moreover, pinocembrin reversed DSS-caused short-chain fatty acid loss and increased the intestinal flora diversity evaluated using 16S rRNA phylogenetic sequencing. Collectively, the evidence suggested a remarkable protective effect of pinocembrin in the prevention of colitis. In another research, Yue et al. (2020) probed the mechanism of pinocembrin against UC. The results showed that pinocembrin relieved DSS-treated UC in murines and ameliorated the intestine injury, diarrhoea and rectal bleeding. The anti-UC mechanism of pinocembrin may be connected with perfecting the disordered intestinal flora composition, restraining TLR4/MD2/NF-κB signal cascades, and maintaining intestine barrier integrality. Overall, pinocembrin could be a viable candidate for the medication of colitis.

## Conclusion and perspectives

**FIGURE 3:**
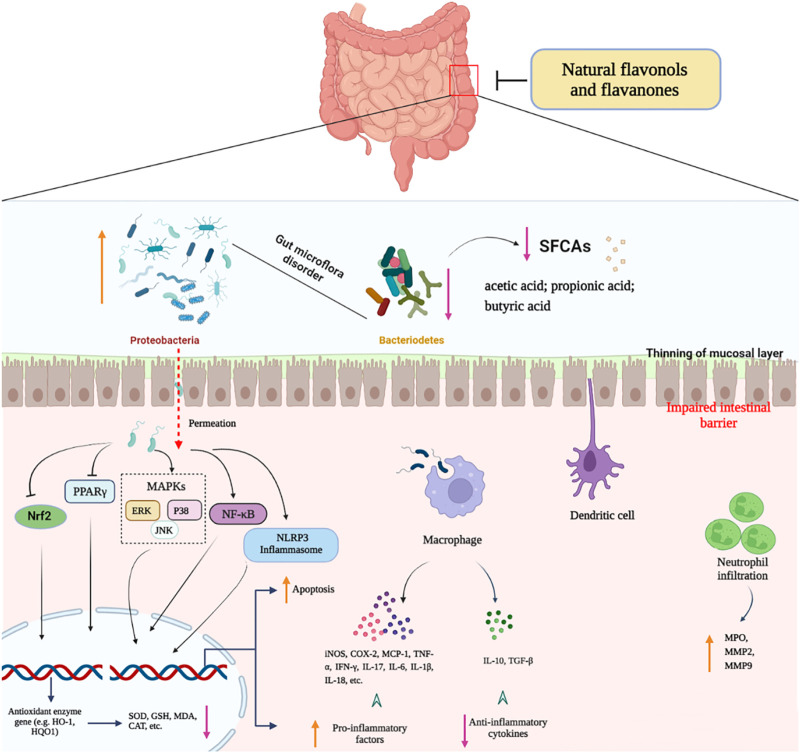
Molecular mechanisms of natural flavonols and flavanones in the therapy of colitis.

This paper summarised the beneficial roles of natural flavonols and flavanones in fighting colitis, and explored the underlying anti-UC mechanisms. Evidence revealed that natural flavonols and flavanones significantly ameliorated the symptoms of UC in multiple animal and cell models by modulating gut microflora, causing the restoration of gut barrier function, and reducing inflammatory reactions, oxidative stress, and apoptosis (Figure 3). Their therapeutic roles were primarily connected with the excitation of Nrf2/HO-1 path and suppression of various signaling comprising NF-κB, MAPKs, STAT3, and NLRP3 inflammasome, indicated that these naturally occurring flavonols and flavanones are promising and efficient candidate medications for colitis. However, further research is required to determine the potential molecular mechanisms of most flavonols and flavanones, and there is currently no evidence from clinical trials to support their practical effects in colitis sufferers. In order to facilitate the possible use of these flavonols and flavanones for colitis treatment in the near future, more effort will be committed to investigating the molecular mechanisms as well as the long-term therapeutic benefits in clinical trials.

## Abbreviations

AA acetic acid; Akt protein kinase B; ALP alkaline phosphatase; AMPK AMP-activated protein kinase; AP alkaline phosphatase; Bcl-2 B-cell lymphoma 2; BMDMs bone marrow-derived macrophages; CAT catalase; Cl cleaved; COX-2 cyclooxygenase 2; CXCL1 chemokine (C-X-C motif) ligand 1; DAI disease activity index; DNMT-1 DNA methyltransferase 1; DSS dextran sodium sulfate; ERK extracellular signal-regulated protein kinase; Foxp3 forkhead box P3; Gli1 Glioblastoma-1; GM-CSF granulocyte macrophage-colony stimulating factor; GSH glutathione; GSK-3β glycogen synthase kinase-3β; HMGB1 high mobility group box protein 1; HO-1 heme oxygenase-1; ICAM-1 intercellular adhesion molecule-1; Ig immunoglobulin; IκBα inhibitor κBα; IL interleukin; iNOS inducible nitric oxide synthase; JNK c-Jun N-terminal kinase; KC keratinocyte-derived chemokine; LDH lactate dehydrogenase; LPL lipoprotein lipase; LPS lipopolysaccharide; MAPK mitogen-activated protein kinases; MCP-1 monocyte chemotactic protein-1; MKRN1 makorin ring finger protein 1; MLKL mixed lineage kinase domain-like; MMP matrix metalloproteinase; MPO myeloperoxidase; NF-κB nuclear factor-kappa B; NLRP3 NOD-like receptor protein 3; NOS2 nitric oxide synthase 2; NPSH non-protein sulphadryls; Nrf2 nuclear factor erythroid 2-related factor 2; PCNA proliferating cell nuclear antigen; PGE2 prostaglandin E2; PKC protein kinase C; PPAR peroxisome proliferator-activated receptor; p-STAT3 phosphorylated signal transducer and activator of transcription 3; Ptc patched; REG3 regenerating islet-derived 3; ROS reactive oxygen species; RORγt tretinoic-acid-receptor-related orphan nuclear receptor gamma;; SCFAs short-chain fatty acids; SGPP2 sphingosine phosphate-phosphatase 2; SGPT serum glutamic pyruvic transaminase; SGOT serum glutamic oxaloacetate transaminase; Shh sonic hedgehog; Smo smoothened; SOD superoxide dismutase; SphK1 sphingosine kinase 1; TAC total antioxidant capacity; TBARS thiobarbituric acid reactive substances; TFF3 trefoil factor family 3; TGF transforming growth factor; T-GSH total glutathione sulphadryls; TJ tight junction; TJP1 tight junction protein 1; LTB4 leukotriene B4; TLR4 toll-like receptor 4; TNBS 2,4,6-trinitrobenzene sulfonic acid; TNF tumor necrosis factor; Th17 T helper cell 17; Treg regulatory T cell; XO xanthine oxidase.
